# Unmet care needs of advanced cancer patients and their informal caregivers: a systematic review

**DOI:** 10.1186/s12904-018-0346-9

**Published:** 2018-07-23

**Authors:** Tao Wang, Alex Molassiotis, Betty Pui Man Chung, Jing-Yu Tan

**Affiliations:** 1 School of Nursing, The Hong Kong Polytechnic University, Hung Hom, Hong Kong; 2 College of Nursing and Midwifery, Charles Darwin University, Darwin, Australia

## Abstract

**Background:**

This systematic review aimed to identify the unmet care needs and their associated variables in patients with advanced cancer and informal caregivers, alongside summarizing the tools used for needs assessment.

**Methods:**

Ten electronic databases were searched systematically from inception of each database to December 2016 to determine eligible studies. Studies that considered the unmet care needs of either adult patients with advanced cancer or informal caregivers, regardless of the study design, were included. The Mixed Methods Appraisal Tool was utilized for quality appraisal of the included studies. Content analysis was used to identify unmet needs, and descriptive analysis was adopted to synthesize other outcomes.

**Results:**

Fifty studies were included, and their methodological quality was generally robust. The prevalence of unmet needs varied across studies. Twelve unmet need domains were identified in patients with advanced cancer, and seven among informal caregivers. The three most commonly reported domains for patients were psychological, physical, and healthcare service and information. The most prominent unmet items of these domains were emotional support (10.1–84.4%), fatigue (18–76.3%), and “being informed about benefits and side-effects of treatment” (4–66.7%). The most commonly identified  unmet needs for informal caregivers were information needs, including illness and treatment information (26–100%) and care-related information (21–100%). Unmet needs of patients with advanced cancer were associated with their physical symptoms, anxiety, and quality of life. The most commonly used instruments for needs assessment among patients with advanced cancer were the Supportive Care Needs Survey (*N* = 8) and Problems and Needs in Palliative Care questionnaire (*N* = 5). The majority of the included studies investigated unmet needs from the perspectives of either patients or caregivers with a cross-sectional study design using single time-point assessments. Moreover, significant heterogeneity, including differences in study contexts, assessment methods, instruments for measurement, need classifications, and reporting methods, were identified across studies.

**Conclusion:**

Both advanced cancer patients and informal caregivers reported a wide range of context-bound unmet needs. Examining their unmet needs on the basis of viewing patients and their informal caregivers as a whole unit will be highly optimal. Unmet care needs should be comprehensively evaluated  from the perspectives of all stakeholders and interpreted by using rigorously designed mixed methods research and longitudinal studies within a given context.

## Background

According to the World Health Organization (WHO), more than 15 million people will be diagnosed with cancer by 2020 [1]. With the advances in cancer treatments, the illness trajectory and prognosis of cancer have changed, and patients diagnosed with advanced cancer can live for a relatively long period [2, 3]. However, lengthy cancer experience and anticancer treatments make patients suffer from a wide range of problems, such as physical, psychological, emotional, and practical issues [4]. Cancer-related symptoms and patients’ experiences during cancer treatment vary across different cancer stages, and patients at advanced stage commonly experience different symptoms from those with early-stage cancer [5, 6]. Such ‘chronic and uncertain’ conditions pose a challenge to not only the cancer services but also to their informal caregivers [7]. Informal caregivers commonly take care of their loved ones for a long period [8]. The long-term caregiving process is physically and psychologically challenging, particularly when taking care of patients with advanced cancer [9]. Many informal caregivers, including those who do not regard caregiving as a burden, suffer from a wide range of problems, such as sleep disturbance, anxiety, depression, and practical and financial difficulties [10, 11]. Informal caregivers are usually regarded as fellow sufferers alongside patients [12]. Unmet needs of patients can increase the level of caregiver burden [13]. In turn, caregivers’ problems are closely linked with patients’ well-being [14], and unsolved problems or unmet needs of caregivers will not only decrease their own quality of life [15] but also affect the patients’ health outcomes negatively [15]. Informal caregivers and patients with advanced cancer are considered a whole unit in fighting the illness [10].

High-quality and patient-and-family-centered care is needed to address the problems of both the advanced cancer patients and their informal caregivers, including symptom and side effect management, as well as emotional, psychosocial, and spiritual support. All these aspects of support are typically categorized under palliative care [16]. Mismatched healthcare that is inconsistent with patients and caregivers’ needs can increase healthcare expenditure and lead to harmful effects [17]. Therefore, the unmet care needs of patients and informal caregivers should be comprehensively assessed prior to designing and providing tailored palliative care services [18, 19]. Care needs are defined as “the requirement of some action or resource in care that is necessary, desirable, or useful to attain optimal well-being” (Foot, 1996, as cited in Sanson-Fisher, et al., 2000, p.227) [20]. Unmet needs assessment is designed to identify how well and how much their needs have been satisfied or not [21]. An early review [17] summarized the instruments for needs assessment; however, a majority of these instruments have been designed for general patients with cancer (e.g., Supportive Care Needs Survey, SCNS [17]). After the publication of that review, several tools that were specifically designed for advanced cancer patients (e.g., Needs Assessment of Advanced Cancer Patients, NA-ACP [22]) have been developed and used.

An early systematic review [21] published in 2009 analyzed the unmet needs of patients with advanced cancer with nine included studies. Another systematic review [7] with 23 studies reported eight unmet need domains. These two systematic reviews only focused on patients, with limited literature searches in only four databases. Meanwhile, the inclusion criteria were relatively ambiguous in the second review because studies with mixed samples (patients at different cancer stages) were included; moreover, the definition of advanced cancer was not presented [7]. Moreover, neither of the two reviews summarized and reported detailed information regarding the needs assessment tools used, which is important information to allow readers to appreciate the quality and reliability of study results. Furthermore, to date, no systematic review has been conducted to explore the unmet needs of informal caregivers of patients with advanced cancer. Therefore, the current systematic review was carried out to update evidence from previous reviews and provide a more comprehensive picture regarding the unmet needs among patients with advanced cancer and informal caregivers. An intensive literature search was performed on 10 electronic databases, and the inclusion criteria were more specific for advanced cancer diagnosis than those of the previous reviews. This current systematic review also included informal caregivers on the basis of the following concepts: fellow sufferers [12], a whole unit [10], and patient-and-family-centered care that is emphasized by the WHO [16]. Specific objectives of this review included: (1) to identify the unmet care needs and their associated factors in patients with advanced cancer and their informal caregivers, and (2) to summarize needs assessment tools that were used in the included studies.

## Methods

### Search strategies

With consideration of the language expertise of the review authors, English and Chinese databases were included. Ten databases, including PubMed, Cumulative Index to Nursing and Allied Health Literature (CINAHL), EMBase, Cochrane Central Register of Controlled Trials (CENTRAL), PsycINFO, Web of Science, Wan Fang Data, China National Knowledge Infrastructure (CNKI), Chongqing VIP (CQVIP), and Chinese Biomedical Literature Database (CBM), were searched systematically from inception of each database to December 2016. Restrictions regarding study design were not set. The used MeSH terms, key words, and free words included needs assessment, assessment of healthcare needs, unmet needs, neoplasms, advanced cancer, terminal cancer, metastatic cancer, and the forth. Manual searches were also conducted by examining the reference lists of the included studies. Three representative search strategies of this systematic review are listed in Table 1.

**Table 1:** Selected Search Strategies

PubMed	
#1	Search ((((“needs assessment”[MeSH Terms]) OR “needs assessment”[Title/Abstract]) OR “assessment of healthcare needs”[Title/Abstract]) OR “assessment of health care needs”[Title/Abstract]) OR “unmet needs”[Title/Abstract]
#2	Search (((((((“palliative care”[MeSH Terms]) OR “palliative medicine”[MeSH Terms]) OR “hospice care”[MeSH Terms]) OR “supportive care”[Title/Abstract]) OR “palliative nursing”[Title/Abstract]) OR “palliative care nursing”[Title/Abstract]) OR “terminal care”[Title/Abstract]) OR “hospice nursing care”[Title/Abstract]
#3	Search (((((“neoplasms”[MeSH Terms]) OR “advanced cancer”[Title/Abstract]) OR “terminal cancer”[Title/Abstract]) OR “metastatic cancer”[Title/Abstract]) OR “tumor”[Title/Abstract]) OR “cancer”[Title/Abstract]
#4	#1 AND #2 AND #3
CINAHL	
#1	TI needs assessment OR TI assessment of healthcare needs OR TI assessment of health care needs OR TI unmet needs
#2	AB needs assessment OR AB assessment of healthcare needs OR AB assessment of health care needs OR AB unmet needs
#3	AB palliative care OR AB palliative medicine OR AB hospice care OR AB supportive care OR AB palliative nursing OR AB palliative care nursing OR AB terminal care OR AB hospice nursing
#4	TI palliative care OR TI palliative medicine OR TI hospice care OR TI supportive care OR TI palliative nursing OR TI palliative care nursing OR TI terminal care OR TI hospice nursing
#5	TI neoplasms OR TI tumor OR TI cancer OR TI advanced cancer OR TI terminal cancer OR TI metastatic cancer
#6	AB neoplasms OR AB tumor OR AB cancer OR AB advanced cancer OR AB terminal cancer OR AB metastatic cancer
#7	#1 OR #2
#8	#3 OR #4
#9	#5 OR #6
#10	#7 AND #8 AND #9
EMBase	
#1	‘needs assessment’/exp
#2	‘needs assessment’:ab,ti OR (assessment:ab,ti AND of:ab,ti AND healthcare:ab,ti AND needs:ab,ti) OR (assessment:ab,ti AND of:ab,ti AND health:ab,ti AND care:ab,ti AND needs:ab,ti) OR ‘unmet needs’:ab,ti
#3	#1 OR #2
#4	‘palliative care’:ab,ti OR ‘palliative medicine’:ab,ti OR ‘hospice care’:ab,ti OR ‘supportive care’:ab,ti OR ‘palliative nursing’:ab,ti OR ‘terminal care’:ab,ti OR ‘hospice nursing’:ab,ti
#5	‘palliative nursing’/exp
#6	#4 OR #5
#7	‘advanced cancer’/exp
#8	‘neoplasm’/exp
#9	‘advanced cancer’:ab,ti OR (terminal:ab,ti AND cancer:ab,ti) OR (metastatic:ab,ti AND cancer:ab,ti) OR neoplasm:ab,ti OR cancer:ab,ti OR tumor:ab,ti
#10	#7 OR #8 OR #9
#11	#3 AND #6 AND #10

### Study identification and data extraction

Duplications were identified and eliminated through a reference management software (NoteExpress). Titles and abstracts of the remaining studies were screened independently by two review authors (WT and TJY), and full text of potentially eligible studies were subsequently located for further screening. Studies satisfying the following inclusion criteria were included: (1) studies that included either adult (≥18 years old) patients with advanced cancer^1^ or adult informal caregivers of patients with advanced cancer; (2) studies that reported data in terms of unmet care needs^2^ or concerns that are directly linked to the unmet care needs of patients with advanced cancer and/or their informal caregivers, regardless of the study design; and (3) accessible full texts were published in peer-reviewed journals. Exclusion criteria were: (1) studies with mixed sample of patients with cancer at any cancer stage (except those patients with advanced cancer who were analyzed separately); (2) studies solely focusing on quality of life [21], satisfaction with healthcare services, care service utilization, or presence of symptoms/problems; (3) studies focusing on instrument development, translation, or evaluation; and (4) conference articles with only abstracts, editorial comments, guidelines, policies, or treatment recommendations. Data were extracted by two independent review authors. These data included information regarding the first author of the study, year of publication, country of origin, research setting, research design, sampling approach, sample size, need assessment methods (interview or other instruments), prevalence of unmet needs, and related factors for unmet needs. Any disagreement was settled and discussed by the two other review authors (CPM and AM).

### Methodological quality appraisal

The methodological quality of included studies was assessed by two review authors (WT and TJY) independently with the Mixed Methods Appraisal Tool (MMAT) [25]. This tool is highly efficient; it takes approximately 14 min to evaluate one study [25] with robust consistency among reviewers (intraclass correlation = 0.72 [25]); MMAT is specifically designed to assess the quality of either quantitative or qualitative studies. Four different quality criteria for qualitative studies and different types of quantitative studies, including randomized control trials, quantitative nonrandomized trials, and quantitative descriptive studies, were used [25]. Each criterion was graded as 0 (unmet) or 1 (meet), and the global score of each study was calculated from 0 to 4 (0 = no criterion satisfied, 1 = satisfied one criterion, 2 = satisfied two criteria, 3 = satisfied three criteria, and 4 = satisfied all four criteria). When any disagreement occurred, the review authors conducted a group discussion to reach final agreement.

### Data analysis

Content analysis [26] was used to identify the unmet need domains of patients with advanced cancer and informal caregivers across quantitative and qualitative studies. A priori content categories of patients with advanced cancer were determined on the basis of previous studies; these categories included health system and information, patient care and support, activities of daily living (ADL), physical, psychological, financial, and spiritual [7]. With regards to informal caregivers, five content categories were determined on the basis of a previous review [10]; these categories included cancer care services, informational, psychological, spiritual, and social needs. Data of the included studies were compared, combined, and clustered with respect to those domains for patients and informal caregivers. Terms, such as instrumental and personal care, were included in the ADL domain because they were frequently mentioned in several North American studies [21]. Summative content analysis was used to identify and extract new categories within content not covered by previous domains. The approach of descriptive analysis was used for the prevalence of unmet needs due to the significant heterogeneity of the included studies [27]. Variables associated with patients and informal caregivers’ needs and used instruments were analyzed through descriptive approach.

## Results^3^

### Characteristics of included studies

**Fig. 1:**
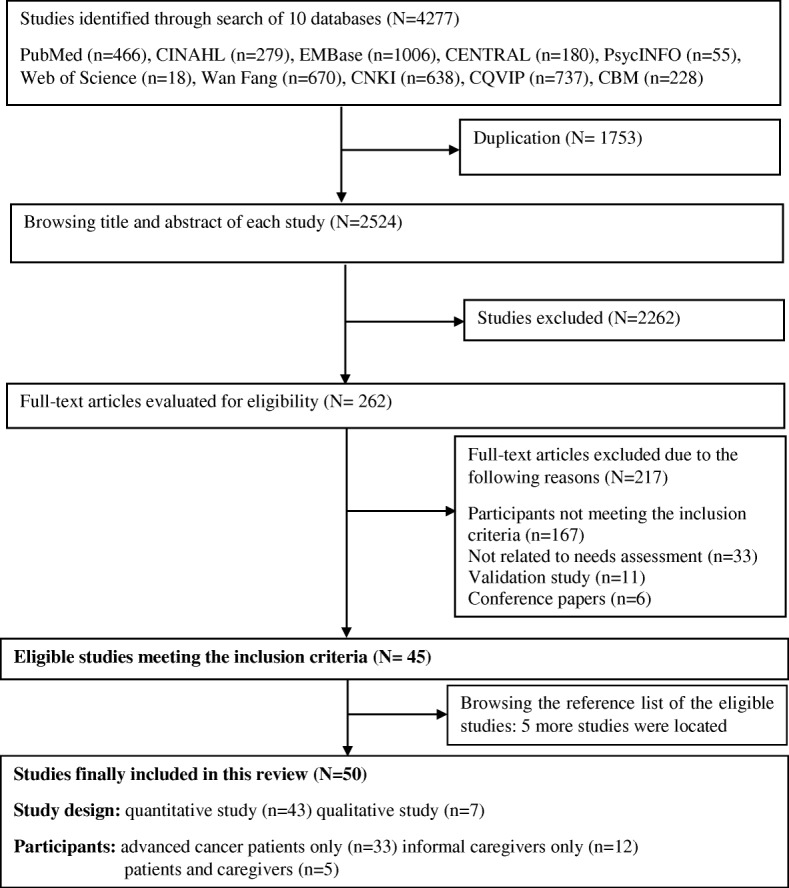
Flow chart of study selection

Among the 4277 potentially eligible studies, 45 studies were included. After screening the reference lists, five other eligible studies were retrieved. Finally, 50 studies [6, 9, 28–75] (5 published in Chinese and 45 in English language) were included in this review **(**Fig. 1**)**. The majority of the studies (43/50) used quantitative study designs, with 42 surveys (1 longitudinal survey [75] and 41 cross-sectional surveys) and 1 [6] pre-post intervention study (only baseline data were used in this review). The seven other studies [48, 49, 57, 62, 71–73] were qualitative designs with individual in-depth interviews and/or focus group. Among the 50 included studies, 33 studies investigated the unmet needs of patients with advanced cancer only, with 31 out of 33 studies from the perspective of patients, one study from the perspective of informal caregivers, and one from the perspectives of both patients and informal caregivers. Twelve studies [9, 30, 32, 35, 39, 40, 49, 51, 52, 57, 62, 64] explored the unmet needs of informal caregivers, and five other [48, 56, 59, 63, 67] studies investigated the unmet needs of patients with advanced cancer and their informal caregivers. With regards to sample sources, six studies [32, 40, 45, 46, 49, 61] reported no information regarding the recruitment setting, while in the remaining studies patients, and/or caregivers were mainly recruited from outpatient departments (*n* = 16), inpatient departments (*n* = 11), home/home-based care units (*n* = 10), and mixed settings (*n* = 7). In terms of cancer sites, 29 studies focused on patients with mixed cancer site and/or their caregivers, 11 studies focused on specific patients with cancer and/or caregivers (3 studies on prostate cancer [57, 69, 73], 5 studies on breast cancer [41, 48, 58, 60, 75], and three on lung cancer [35, 42, 71]), while 10 other studies [47, 50, 51, 53, 59, 64, 66, 68] reported no information about cancer types. The diagnostic criteria of advanced cancer were presented in 13 studies (13/50), with five studies [6, 30, 31, 60, 61] adopting the criteria of cancer with metastasis, and seven studies [9, 41, 42, 45, 58, 63, 75] using the stage III/IV criterion according to TNM staging system. With regards to geographic distribution, nine studies were conducted in the USA [38, 40, 46, 49, 52, 57, 59, 70, 74], seven were in mainland China (six of which were conducted in Shanghai) [9, 53, 63–67], five in Australia [6, 54, 55, 60, 68], five in the Netherlands [29–31, 34, 44], four in Canada [47, 50, 56, 73], three in Japan [33, 39, 41], three in Taiwan [35, 42, 62], two in the UK [69, 71], two in Denmark [45, 72], two in Hong Kong [58, 75], and one each in Italy [28], France [61], South Korea [32], Spain [37], Indonesia [36], Czech Republic [43], India [51], and Bangladesh [48]. Characteristics and main findings of all included studies are presented in Table 2.

### Quality of the included studies

The methodological quality of the included studies was generally robust, with 17 and 18 studies satisfying all four criteria (34%) and three of the four criteria (36%), respectively. The prominent weaknesses of 43 quantitative studies were poor sampling strategy and low response rate. The response rates of 16 studies [32, 33, 37, 39, 40, 43, 47, 52–56, 61, 63, 68, 74] were lower than 60%, and 14 studies [30–32, 38, 42, 43, 51–54, 67, 68, 70, 74] failed to report the sampling method, sampling procedure, or sample size justification. Among the seven other qualitative studies, three studies (3/7, 42.9%) [49, 62, 73] failed to interpret how findings were related to the study context, and two studies (2/7, 28.6%) [57, 73] provided no explanation on how the research process was influenced by the researchers. The overall quality score of each study is presented in the first column of Table 2.

**Table 2:** Characteristics and Main Findings of the Included Studies

Author, Year & QS	Country/ Region	Setting	Study Design	Participant	Diagnosis	Response Rate	Data Collection Method/ Instrument & Findings
Studies Regarding Advanced Cancer Patients (*n* = 33)							
S1 [28]: Morasso G, et al., 1999, QS:3	Italy	Inpatients	Semi-structured interview survey	Sampling: Random sampling Sample size: 94 Age (yr): 64.8 ± 11.1 Gender: 38/89 (F)	Terminal cancer patients (mixed cancer sites)	89/94 (94.7%)	Interviews guide: 5 domains and 41 items: “physiological needs”, “safety needs”, “loved and belonging needs”, “self-esteem needs” and “self-fulfillment needs” (p.404)
							Unmet needs (p.406): 1) symptoms control (62.8%), 2) occupational functioning (62.1%), 3) emotional support (51.7%), 4) Nutrition (43.2%), 5) sleep (37.1%), 6) self-fulfillment (32.5%), 7) communication (27.7%), 8) information (25.0%), 9) personal care (14.6%), 10) financial support (14.1%) and 11) emotional closeness (13.8%)
S2 [6]: Waller, et al., 2012, QS: 4	Australia	Outpatients	Multiple time points pre-post intervention study ^a^	Sampling: unclear (219/613) Sample size: 219 Age (yr): 66.1 ± 10.7 Gender: 91/195 (F)	Advanced cancer patients (extensive local, regional or metastatic) (mixed cancer sites)	195/219 (89.0%)	Supportive Care Needs Survey (SCNS-SF34): 5 domains and 34 items Needs Assessment for Advanced Cancer Patients (NA-ACP): only used 6 items on spiritual needs
							Moderate-to-high unmet needs: 1) “not being able to do the things you used to do” (33.0%), 2) “concerns about the worries of those close to you” (27.9%), 3) “lack of energy, tiredness” (26.2%), 4) “work around the home” (23.0%), 5) “uncertainty about the future”(21.4%), 6) “pain” (20.9%), 7) “worry that results of treatment are beyond your control” (19.4%), 8) “fears about the cancer spreading” (18.8%), 9) “felling unwell a lot of the time”(17.3%), and 10) “anxiety” (15.3%)
S3 [29]: Teunissen, SC, et al., 2006 QS: 3	Netherla-nds	Inpatients	Structured interview survey	Sampling: unclear Sample size: 181 Age (median, yr): 18–79 Gender: 101/181 (F)	Advanced cancer patients (mixed cancer sites)	181/181 (100%)	Structured interview with a standard list: 4 domains: emotional needs, social needs, spiritual needs, and functional needs. (p.153) Each item including 2 parts: 1) if the issue is a “problem”; 2) actual wishes to receive professional support were labelled as palliative care needs. (p. 153)
							Unmet needs: 1) functional support (62.4%), 2) support in coping (57.5%), 3) emotional support (53.1%), 4) support of informal caregivers (34.3%), 5) spiritual support (7.7%), 6) co-ordination of care (9.9%), 7) relational support (9.9%), and 8) support in communication (7.7%).
S5 [31]: Osse BHP, et al., 2005, QS: 3	Netherla-nds	Home-based	Questionnaire survey	Sampling: unclear? Sample size: 112 Age (yr): 58 ± 12.3 (30–87) Gender: 66/94 (F)	Distant metastatic cancer (mixed cancer sites)	94/112 (84.0%)	Problems and Needs in Palliative Care questionnaire (PNPC): 10 domains and 90 items
							Top 10 unmet needs: 1) “difficulty coping with the unpredictability of the future” (25%), 2) “fear of metastases” (25%), 3) “fear of physical suffering” (24%), 4) “experiencing difficulties in remembering what was told” (24%), 5) “difficulties to accept the disease” (23%), 6) “extra expenditure because of disease” (23%), 7) “fear of death” (21%), 8) “frustrations because I can do less than before” (20%); 9) “experiencing loss of control over one’s life” (19%); 10) “fear of treatments” (19%)
S7 [33]: Hasegawa, et al., 2016 QS: 3	Japan	Inpatients	Questionnaire survey	Sampling: random sampling Sample size: 45 Age (yr): 66.6 ± 9.8 Gender: 21/45 (F)	Advanced cancer patients (mixed cancer sites)	NR	Supportive Care Needs Survey (SCNS-SF34): 5 domains and 34 items Hospital Anxiety and Depression Scale (HADS) Functional Independence Measure (FIM)
							Top 10 Moderate-to-high unmet needs: 1) “Being informed about things you can do to help yourself to get well” (51.1%); 2) “Having one member of hospital staff with whom you can talk to about all aspects of your condition, treatment, and follow-up” (51.1%); 3) “Concerns about the worries of those close to you”(44.1%); 4) “Anxiety”(41.8%); 5) “Not being able to do the things you used to do” (37.2%); 6) “Feeling down or depressed” (37.2%); 7) “Being treated like a person not just another case” (34.8%); 8) “Hospital staff acknowledging, and showing sensitivity to, your feelings and emotional needs” (34.8%); 9) “Hospital staff attending promptly to your physical needs” (34.8%); 10) “Feelings of sadness” (32.5%); 11) “Feelings about death and dying”; (32.5%); 12) “Reassurance by medical staff that the way you feel is normal” (32.5%); 13) “Learning to feel in control of your situation” (32.5%);
S8 [34]: Uitdehaag MJ et al., 2015 QS: 4	Netherla-nds	Outpatients	Questionnaire survey	Sampling: consecutive sampling Sample size: 57 Age (yr): EC: 65 ± 11.8 PBC: 64 ± 12.2 Gender: EC: 2/24 (F) PBC:10/33 (F)	Incurable EC or PBC cancer patients	57/90 (63%), with 24 EC and 33 PBC	Problems and Needs in Palliative Care questionnaire (PNPC): 9 domains and 90 items EORTC QLQ-OES18 EORTC QLQ-PAN26
							Unmet needs: EC: 1) “fatigue” (21%); 2) “frustration can do less than usual” (21%); 3) “shortness of breath” (17%) PBC: 1) “fear of physical suffering” (34%), 2) “lack of written information” (28%), 3) “fatigue” (22%).
S10 [36]: Effendy, C, et al., 2014 QS: 2	Indonesia	Outpatients	Questionnaire survey	Sampling: unclear Sample size: 180 Age (yr): Indonesian: 49.3 ± 10.7 Netherlands: 58 ± 12.3 Gender: Indonesian: 133/180 (F) Netherlands: 66/94 (F)	Advanced cancer (mixed cancer sites)	NR	Revised Problems and Needs in Palliative Care questionnaire-short version (PNPC-sv,24 items): adjusted within Indonesian context and deleted 9 items, and 24 items were maintained
							Unmet needs: Physical: sweating (76.2%), sexuality (75%), short of breathless (67.3%), pain (66.4%) Autonomy: “difficulties in finding someone to talk to” (82.8%); Psychological: “difficulties showing emotions” (84.4%) Spiritual: “difficulties about the meaning of death” (85.4%) Financial: “extra expenses because of the disease” (72%)
S11 [37]: Vilalta, A, et al., 2014 QS: 3	Spain	Outpatients	Questionnaire survey	Sampling: unclear Sample size: 50 Age (yr): Mean 60.9 (33–81) Gender: 19/50 (F)	Advanced cancer (mixed cancer sites)	NR	Self-designed questionnaire for spiritual needs:11 domains and 28 items
							Top 10 spiritual needs (p. 594): 1) “to be recognized as a person until the end of life” (8.6 ± 1.3); 2) “the need for truth” (8.3 ± 2.7); 3) “to reinterpret life” (6.2 ± 1.9); 4) “to look for a meaning to existence” (5.7 ± 2.5); 5) “the need for hope” (5.7 ± 3.5); 6) “to see life beyond the individual” (5.2 ± 2.5); 7) “the need for religious expression” (4.9 ± 2.5); 8) “the needs for continuity and an afterlife” (4.0 ± 2.0); 9) “the need for freedom and to be free” (3.8 ± 3.4); 10) “to be free blame and to forgive others” (1.5 ± 2.0).
S12 [38]: Schenker Y. et al., 2014 QS: 3	USA	Outpatients	Questionnaire survey	Sampling: unclear Sample size: 169 Age (yr): 62.3 ± 11.6 Gender: 107/169 (F)	Advanced cancer (mixed cancer sites)	169/272 (62.1%)	Adapted Needs Assessment of Advanced Cancer Patients (NA-ACP): 32 items and 6 domains, without reporting psychological properties
							Unmet needs: 1) symptom (62%); 2) psychological (62%); 3) medical communication/information (39%); 4) daily living (27%); 5) spiritual (23%); 6) social (20%)
S16 [41]: Uchida M, et al., 2011 QS: 4	Japan	Outpatients	Questionnaire survey	Sampling: random sampling Sample size: 85 Age (yr): 58.6 ± 11.9 Gender: 85/87 (F)	Advanced breast cancer patients (stage IV)	85/87 (97.7%)	Supportive Care Needs Survey (SCNS-SF34): 5 domains and 34 items Hospital Anxiety and Depression Scale (HADS) EOERC-QLQ-C30
							Top 10 moderate-to-high unmet needs: 1) “Fears about the cancer spreading” (78.8%); 2) “Worry that the results of treatment are beyond your control” (71.8%); 3) “Concerns about the worries of those close to you” (68.2%); 4) “Having one member of hospital staff with whom you can talk to about all aspects of your condition, treatment and follow-up” (67.1%); 5) “Being informed about things you can do to help yourself to get well” (65.9%); 6) “Anxiety” (65.9%); 7) “Feeling down or depressed” (62.4%); 8) “Uncertainty about the future” (62.4%); 9) “Feeling about death and dying” (62.4%); 10) “Having access to professional counseling if you, family or friends need it” (57.6%);
S17 [42]: Liao YC, et al., 2011 QS: 3	Taiwan	Mixed	Questionnaire survey	Sampling: unclear Sample size: 152 Age (yr): 60.2 ± 11.0 Gender: 73/152 (F)	Advanced lung cancer patients (95.4% stage III-IV or extensive metastasis)	152/188 (80.9%)	Cancer Needs Questionnaire (CNQ)-Chinese version: 5 domains and32 items Hospital Anxiety and Depression Scale (HADS) Symptom Severity Scale (SSS)
							Items of highest unmet needs by each domain: 1) “things helping self get well” (65.8%), 2) “cancer remission” (63.8%), 3) “benefit and side-effects of treatment” (63.8%), 4) “test results as soon as possible” (62.5%); 5) “dealing with fears about disease spreading and return” (40.2%), 6) “doctor acknowledges and shows sensitivity to your feelings and emotional needs” (39.5%), 7) “dealing with lack of energy and tiredness” (28.3%)
S18 [43]: BUŽGOVÁ, et al., 2014 QS: 2	Czech Republic	Inpatients	Questionnaire survey	Sampling: unclear Sample size: 93 Age (yr): 61.6 ± 16.8 Gender: 41/93 (F)	Advanced cancer (mixed cancer sites)	NR	Patient Needs Assessment in Palliative Care (PNAP): 5 domains and 42 items Hospital Anxiety and Depression Scale (HADS) EOERC-QLQ-C30
							Items of highest unmet needs by each domain: 1)Spiritual: “attending religious services or other ceremonies” (44%); 2) Autonomy: “continue my usual activities” (38%); 3) Social: “being financially secure” (27%); 4) psychological: “fear of dependence on help from others” (30%); 5) physical: “fatigue” (30%);
S19 [44]: Voogt E, et al., 2005 QS: 4	Netherlands	Home-based	Questionnaire survey	Sampling: unclear Sample size: 128 Age (yr): 63.6 ± 10.5 Gender: 66/128 (F)	Advanced cancer (mixed cancer sites)	128/192 (66.7%)	Problems and Needs in Palliative Care questionnaire (PNPC): used the 12 items on information needs Hospital Anxiety and Depression Scale (HADS) Utrecht Coping List to measure disease-specific coping
							Unmet information: 1) complementary care (93%); 2) alternative medicine (86%); 3) euthanasia: (83%); 4) care settings (78%); 5) Sexuality and cancer (72%); 6) psychological care (71%); 7) cause of cancer (65%); 8) food and diet (44%); 9) helpful devices (33%); 10) organizations that offer help (32%); 11) expected physical (20%); 12) treatment options and side effects (4%)
S20 [45]: Johnsen AT, et al., 2013 QS: 4	Denmark	NR	Questionnaire survey	Sampling: random sampling Sample size: 977 Age (yr): mean 64 Gender: 547/977 (F)	Advanced cancer with mixed sites (95% at stage III/ IV)	977/1630 (60%)	3-Levels-of-Needs Questionnaire (3LNQ):12 items
							Unmet needs: 1) fatigue (35%); 2) physical activities (32%); 3) work and daily activities (29%); 4) worry (31%); 5) sexuality (28%); 6) pain (23%); 7) concentration (25%); 8) depression (24%); 9) dyspnea (19%); 10) nausea (12%); 11) lack of appetite (13%); 12) difficulties with family life and contact with friends (11%)
S21 [46]: Houts P, et al., 1988 QS: 4	USA	NR	Semi-structured interview survey (retrospective)	Sampling: stratified random sampling Sample size: 433 Age (yr): ≥20y Gender: unclear	Caregivers of terminal cancer (mixed cancer sites)	433/515 (84.0%)	Self-designed questionnaire of needs in cancer patients, including 14 areas: physical, activities of daily lives, reaction to treatment, nutrition, emotional, life purpose, social, family, financial, insurance, getting health care, medical staff, home health care, and transportation (p. 629)
							Unmet needs: 1) activities of daily lives (42%); 2) emotional (21%); 3) physical (21%); 4) insurance (19%); 5) financial (15%); 6) medical staffs (20%)
S22 [47]: Khan L, et al., 2012 QS: 3	Canada	Outpatients	Questionnaire survey	Sampling: unclear Sample size: 40 (patients = 20, caregivers = 20) Age (yr): Patients: unclear Caregivers: unclear Gender: unclear	Advanced cancer patients and their caregivers (cancer site unclear)	NR	Problems and Needs in Palliative Care- short version (PNPC-sv): 8 domains and 33 items
							Patients’ unmet needs from their own perspectives: 1) “doing light housework” (25%); 2) “pain” (25%), 3) “fatigue” (25%), 4) “personal transportation” (22.2%); 5) “sleeping problems” (21.1%); 6) “body care, washing, dressing, or toilet” (20%); 7) “fear of metastases” (17.6%); 8) “pricking or numb sensation” (16.7%); 9) “experiencing loss of control over one’s life” (16.7%), 10) “fear of physical suffering” (16.7%) Patients’ unmet needs from caregivers’ perspectives: 1) “sexual dysfunction” (100%);2) “problems in relationship with life companion” (100%); 3) “finding others not receptive to talking about the disease” (100%); 4) “difficulties to show emotions” (100%), 5) “difficulties to be of avail for others” (100%), 6) “difficulties to accept the disease” (100%), 7) “extra expenditures because of the disease” (100%), 8) “loss of income because of the disease” (100%), 9) “pain”(35%), 10) “fear of physical suffering” (29.4%)
S25 [50]: Fitch MI, 2012 QS: 4	Canada	Outpatients	Questionnaire survey	Sampling: convenience sampling Sample size: 69 Age (yr): mean 65y (35-84y) Gender: 34/69 (F)	Advanced cancer patients (cancer sites unclear)	69/106 (65.1%)	Adapted Supportive Care Needs Survey (SCNS): 7 domains and 61 items: information, physical symptoms, psychological, emotional, spiritual, social, and practical, Cronbach’s α = 0.35–0.81
							Unmet needs in terms of issues reported by 50% patients: 1) “pain” (63.5%); 2) “fear of pain” (62.9%); 3) “lack of energy” (52.8%); 4) “fear about physical disability or deterioration” (50%); 5) “fear about cancer spreading” (51.4%;); 6) “not being able to do things you used to” (46.9%); 7) “decreased appetite” (47.4%); 8) “feeling unwell” (44.7%); 8) “feeling down or depressed” (30%), 9) “not being able to work around at home” (44.2%); 10) “concerns about the worries of those close to you”(29.4%)
S28 [53]: Deng D, et al. 2015 QS: 2	China	Home-based	Interview survey	Sampling: unclear Sample size: 107 Age (yr): mean 57y (18-87y) Gender: 58/107 (F)	Advanced cancer patients (cancer sites unclear)	NR	Guided life review (2–3 times in-depth interview)
							Three expectations (spiritual needs) (p.728): 1) have a nice day without pain (14.3%) 2) wish family health and happiness (37.6%) 3) fulfill patients’ dreams (witness future family events, company of their families, etc.)(45.8%)
S29 [54]: Rachakonda K, et al., 2015 QS: 1	Australia	Inpatients	Questionnaire survey	Sampling: unclear Eligible sample: unclear Sample size:75 Age (yr): 68 ± 12 Gender: 32/75 (F)	Advanced cancer patients (mixed cancer sites)	NR	Needs Assessment of Advanced Cancer Patients (NA-ACP): 7 domains and 132 items
							Items of highest unmet needs by each domain: 1) symptom “dealing with lack of energy or tiredness” (30.7%); 2) psychological “coping with frustration at not being able to do the things you used to do” (24.3%); 3) daily livings “getting assistance with preparing meals” (12%); 4) social “receiving emotional support from friends and family” (12.2%); 5) medical information and communication (9.3–14.9%), “getting information about non-conventional treatments” (14.9%); 6) financial “paying the non-medical costs of your illness”; (17.3%); 7) spiritual “being able to choose the place where you want to die” (11%).
S30 [55]: Rainbird K, et al. 2009 QS: 3	Australia	Home-based	Questionnaire survey	Sampling: unclear Sample size: 246 Age (yr): 61 ± 11.9 Gender: 131/246 (F)	Advanced cancer patients (mixed cancer sites)	246/418 (59%)	Needs Assessment of Advanced Cancer Patients (NA-ACP): 7 domains and 132 items
							Items of highest unmet needs by each domain: 1) symptom (15–22%)’ “dealing with loss of appetite” (22%); 2) psychological (39–40%), “coping with fears about the caner spreading” (40%) and “coping with frustration at not being able to do the things you used to do” (40%); 3) daily livings (10–30%), “dealing with doing work around the house” (30%); 4) social (10–13%), “being able to express feeling with friends and/or family” (13%); 5) medical information and communication (31–35%), “getting information about factors, which could influence the course of the cancer” (35%); 6) financial (11–12%), “dealing with concerns about your financial situation” (12%); 7) spiritual (11–15%), “being able to choose the place where you want to die” (15%)
S33 [58]: Au A, et al., 2013, QS: 4	Hong Kong	Outpatients	Questionnaire survey	Sampling: consecutive sampling Sample size: 198 Age (yr): 53.4 ± 9.74 Gender: 198/198 (F)	Advanced breast cancer patients (stage III/IV)	198/220 (90%)	Chinese version of Supportive Care Needs Survey (SCNS-SF33-C): 4 domains and 33 items: physical and daily living, psychological, sexuality, health system, information and patient support (HSIPS) Hospital Anxiety and Depression Scale (HADS) Memorial Symptom Assessment Scale Short-Form (MSAS-SF) Chinese Patient Satisfaction Questionnaire
							Top 10 moderate-to-high unmet needs: 1) “Having one member of hospital staff with whom you can talk to about your concerns” (63.7%); 2) “informed about cancer is under control or diminishing” (61.6%); 3) “Informed about things you can do to get well” (58.6%); 4) “Informed about your test results” (51%); 5) “Given written information” (46.9%); 6) “given information about aspects of managing illness and side-effects at home” (39.9%); 7) “adequately information about the benefits and side-effects of treatments” (39.3%); 8) “given explanations of those tests for which you would like explanations” (36.9%); 9) “being treated like a person” (35.4%); 10) “more choice about cancer specialists” (31.8%)
S35 [60]: Aranda S, et al., 2005 QS: 4	Australia	Outpatients	Questionnaire survey	Sampling: consecutive sampling Sample size: 105 Age (yr): (34–85, median 57) Gender: 105/105(F)	Metastatic breast cancer	105/172 (61%)	Supportive Care Needs Questionnaire (SCNQ): 5 domains and 59 items
							Moderate to high unmet needs: 1)Psychological needs (24–41%): “concerns about the worries of those close to you” (41%), “uncertainty about the future” (38%), etc. 2)Information needs (26–41%): “informed about things you can do to help yourself get well” (41%), “one member of hospital staff with whom you can talk” (32%), etc. 3)Physical and daily living needs (25–28%): “pain” (28%), “not being able to do the things you used to” (25%).
S36 [61]: Lelorain S, et al., 2015 QS: 2	France	NR	Questionnaire survey	Sampling: consecutive sampling Sample size: 201 Age (yr): mean 62 Gender: 146/201 (F)	Metastatic cancer (mixed cancer sites)	NR	Adapted Supportive Care Needs Survey (SCNS): 2 domains and 13 items: psychological dimension, and staff-related dimension. Seven-point scale (1–7): 1 = no need at all, 7 = a total need of help
							Unmet needs: 1) psychological needs: “being informed about things you can do to help yourself to get well” (3.83 ± 2.24), etc. 2) staff-related needs: “being informed about your test results as soon as feasible”(3.44 ± 2.27), etc.
S40 [65]: Gu WJ, et al., 2015 QS: 3	Shanghai, China	Inpatients	Questionnaire survey	Sampling: convenience sampling Sample size: 134 Age (yr): 75.9 ± 10.5 Gender: 62/134 (F)	Advance cancer (mixed cancer sites)	134/134 (100%)	Self-designed questionnaire for needs including 4 parts (26 items) (p. 2656): basic information, quality of life, health care service needs and attitudes towards disease and death
							Needs: 1) psychological (47%); 2) daily living (31.3%); 3) spiritual (13.4%); 4) families’ support and accompany (67.9%); 5) needs of volunteers (18.7%); 6) friends’ support and accompany (59%)
S41 [66]: Huang J, et al., 2008 QS: 3	Shanghai, China	Home-based	Questionnaire survey	Sampling: random sampling Sample size: 113 Age (yr): 58.31 ± 8.7 Gender: 54/113 (F)	Advance cancer (cancer sites unclear)	113/116 (97.4%)	Self-designed questionnaire for needs including (items: not described)
							Needs on community wards: (pp. 34–35) 1) treatment care like transfusion, injection (77%); 2) pain (46.9%); 3) constipation, nausea (45.1%); 4) information about disease (37.2%) and rehabilitation (32.7%), psychological like anxiety (38.9%), sense of fear (20.4%). Needs on home-based care: 1) treatment care like transfusion, injection (71.7%); 2) regular health assessment (43.4%); 3) knowledge about nutrition (31.0%) and care skills (23.9%), pain (36.3%), communication (28.3%). Needs on day care center: 1) treatment care like transfusion, injection (69%); 2) regular health assessment (42.5%); 3) information and education (28.3%); 4) communication (18.6%); 5) nutrition (38.9%)
S43 [68]: Waller A, et al., 2012 QS: 2	Australia	Mixed	Multi-center questionnaire survey	Sampling: unclear Sample size: 219 patients NAT: PD-Cs were completed on 120 patients Age (yr): 66.1 ± 10.7 Gender: 90/198 (F)	Advance cancer (cancer sites unclear)	36%	Needs Assessment Tool: Progressive Disease-Cancer (NAT: PD-C): 4 sections and 18 items (significant)
							Overall: 80% had at least one concern Patients’ well-being: 1) physical:58% 2) daily living: 29% 3) psychological:19%
S44 [69]: Templeton, H, et al., 2003 QS: 4	UK	Home-based	Structured interview survey	Sampling: unclear Sample size: 90 Age (yr): 71–80 (48.9%) Gender: 90 (M)	Advance prostate cancer	79%	Adapted Toronto Information Needs Questionnaire (TINQ-BC): 5 domains and 29 items
							Unmet needs: 82.2% of the patients need more information: 1) “side effects of treatment” (66.7%); 2) “how to ease side effects of treatment” (64.4%)
S45 [70]: Hwang, S, et al., 2004 QS:3	USA	Mixed	Questionnaire survey	Sampling: consecutive sampling Sample size: 296 Age (yr): median 68 (29–96) Gender: 296 (M)	Advance cancer (mixed cancer sites)	296/312 (94.9%)	14-item unmet needs questionnaire: 5 domains and 14 items
							Unmet needs: 1) physical: 46.1–80%; 2) emotional/social: 10.1–32.5% 3) economic: 6.6–17.3% 4) medical: 12.5–13.6% 5) community: 0–14.3%
S46 [71]: Murray, SA, et al., 2004 QS: 4	UK	Outpatients	Semi-structured interview	Sampling: purposive sampling Sample size: 20 Age (yr): median 65 Gender: unclear	Advance lung cancer	NA	Semi-structured interview, 40mins- 2 h, tape recorded
							Unmet needs: 1) “fear, distress and uncertainty” (p. 41) 2) review “what they had achieved, what still needed to be done before death” (p. 42), and establish themselves as they ‘really’ are (p. 41) 3) “feeling of loss of control” (p. 42) 4) “hard to find hope,” and “questioned their faith wonder why God had not heeded their prayers” (p.42)
S47 [72]: Soelver L, et al., 2014 QS: 4	Denmark	Inpatients	Semi-structured interview	Sampling: open and strategic sampling Sample size: 11 Age (yr): median 71.3 (54–86) Gender: 7/11 (F)	Advance cancer (mixed cancer sites)	NA	Semi-structured interview, 30mins- 1 h
							Unmet needs (pp. 177–180): 1) professionals failed to provide patients timely information; 2) patients experienced that “professionals failed to give much help in terms of physical and emotional burden”; 3) Not being regarded as a person: “lack of dialogue with professionals make patients feel neglected and uncertain in the sense of belonging”; 4) autonomy: “patients wanted to be proactive in problem solving, but did not know how to do”; 5) lack of help for their physical and emotional problem
S48 [73]: Cater N, et al., 2011 QS: 2	Canada	Outpatients	Semi-structured focus group and in-depth interview	Sampling: unclear Sample size: 29 Age (yr): mean 75 (59–88) Gender: 29 (M)	Advance prostate cancer	NA	Semi-structured focus group (90–120 min) and in-depth interview (30–60 min), tape recorded
							Unmet needs (pp. 191–193): 1) function issues: pain, fatigue, side (e. g., urinary incontinence issues, loss of sexual function, etc.); 2) information needs of treatment, medication, side effects and health care service etc.; 3) emotional distress: sadness, anger, frustration and regret which associated with some unsolved issues about diagnosis and treatment decisions.
S49 [74]: Christ G, et al., 1990 QS: 1	USA	Outpatients	Interview survey	Sampling: unclear Sample size: 200 Age (yr): 45–64 (54%) Gender: 62% (F)	Advance cancer (mixed cancer sites)	NR	Structured in-depth telephone interview (30 min)
							Unmet needs (p. 762): 1) personal: 6%; 2) instrumental: 43%; 3) administrative: 38%; 4) medical:18%
S50 [75]: Lam W. W.T, ET AL., 2014 QS: 4	Hong Kong	Outpatients	Questionnaire survey (longitudinal)	Sampling: consecutive sampling Sample size: 228 Age (yr): 53.4 ± 9.79 Gender: 228 (F)	Advance breast cancer (stage III/IV)	228/262 (87.0%)	Supportive Care Needs Survey- Chinese version (SCNS-SF33): 4 domains and 33 items Hospital Anxiety and Depression scale (HADS): 14 items Memorial Symptom Assessment Scale Short-form (MSAS-SF)- Chinese version: 32 items
							Top 10 Moderate-to-high unmet needs: 1) “Having one member of staff with whom you can talk to about all aspects of your condition” (64.5%), 2) “Being informed about cancer which is under control” (60.4%), 3) “Being informed about things you can do to help yourself to get well” (57.4%), 4) “Being informed about your test results as soon as feasible” (50.8%), 5) “Being given written information about the important aspects of your care” (42.3%), 6) “Being adequately informed about the benefits and side effects of treatments before you choose to have them” (42.3%), 7) “Being given explanations of those tests for which you would like explanations” (37.6%), 8) “Being treated like a person not just another case” (34.5%), 9) “Being given information about aspects of managing your illness and side effects at home” (34.2%), 10) “More choice about which cancer specialists you see” (30.5%).
Studies Regarding Informal Caregivers (*n* = 12)							
Author, Year & QS	Region	Setting	Study Design	Participants	Diagnosis	Response Rate	Data Collection Method/ Instrument
S4 [30]: Osse BHP, et al., 2006 QS: 3	Netherlands	Home-based	Questionnaire survey	Sampling: unclear? Sample size: 81 Age (yr): mean 54y (28-78y) Gender: 30/76 (F)	Informal caregivers of mixed advanced cancer patients (distant metastasis)	76/81 (93.8%)	Problems and Needs in Palliative Care questionnaire-caregiver form (PNPC-c): 67 items
							Unmet needs (top 10): 1) “knowing physical signs what I should notice” (25%), 2) “lacking of information in writing” (23%); 3) “fear of an unpredictable future” (22%), 4) “difficulty in coordinating the care of different professionals” (22%), 5) “difficulty in getting access to help from agencies/professional organizations” (22%); 6) “difficulty in getting a second opinion from another doctor” (21%), 7) “how I should handle the patient’s pain” (21%), 8) “extra expenditure because of the disease” (17%), 9) “insufficient adjustment of hospital care to the home situation” (17%), 10) “the possibility to choosing another care provider” (14%) Information needs: information on 1) “the physical problems” (69%), 2) “expectations for the future” (59%), 3) “the possibilities of treatment and side effects” (52%); 4) “euthanasia” (41%); 5) “cause on cancer”(39%), 6) “on nourishment” (37%); 7) “on places and agency that provide help” (30%); 8) “aids to help me” (29%)
S6 [32]: Park SM, et al., 2010 QS: 1	South Korea	NR	Questionnaire survey (retrospective)	Sampling: unclear? Sample size: 1662 Age (yr): not report Gender: 1099/1662 (F)	Informal caregivers of mixed advanced cancer patients (patients died)	1662/4042 (41.4%)	Self-designed needs questionnaire: including 5 domains: 1) symptom management, 2) psychosocial support, 3) financial support, (4) community support, including volunteer assistance, and 5) religious support.. (p.701)
							Unmet needs (p. 703): 1) symptom support (42.8%), 2) financial support (42.7%), 3) psychological support (20.6%), 4) community support (19.7%), and 5) religious support (3.8%)
S9 [35]: Chen SC, et al., 2016 QS: 4	Taiwan	Mixed	Questionnaire survey	Sampling: consecutive sampling Sample size: 166 Age (yr): 49.6 ± 12.0 Gender: 71/166 (F)	Informal caregivers of advanced lung cancer patients	166/190 (87.4%)	1) Partners and Caregivers supportive care needs survey (SCNS-P&C):6 domains and 44 items 2) Numerical rating scale (NRS) (0–10, 0 = no fatigue or sleep disturbance, 10 = worst imaginable): fatigue or sleep disturbance
							Top 10 unmet needs: 1) “Managing concerns about the cancer coming back” (78.3%); 2) “Addressing fears about the person with cancer’s physical or mental deterioration” (72.3%); 3) “Ensuring there is an ongoing case manager to coordinate services for the person with cancer” (71.1%); 4) “Accessing information on what the person with cancer’s physical needs are likely to be” (68.7%); 5) “Accessing information about the person with cancer’s prognosis, or likely outcome” (65.1%); 6) “Accessing information about the benefits and side-effects of treatments so you can participate in decision making about the person with cancer’s treatment” (62.1%); 7) “Obtaining adequate pain control for the person with cancer” (61.5%); 8) “Finding out about financial support and government benefits for you and/or the person with cancer” (60.9%); 9) “Understanding the experience of the person with cancer” (58.5%); 10) “Reducing stress in the person with cancer’s life” (56.1%)
S13 [9]: Cui J, et al., 2014 QS: 4	Shanghai, China	Inpatients	Questionnaire survey	Sampling: convenience sampling Sample size: 649 Age (yr): 49.2 ± 13.18 Gender: 369/649 (F)	Family caregivers of mixed advanced cancer patients (stage IV)	649/700 (95.6%)	Self-designed needs questionnaire: 7 dimensions and 36 items (p. 567) Cronbach’s α = 0.902
							Scores of Needs (p. 567): 1) “maintaining health” (3.48 ± 1.04); 2) “support from professionals” (4.11 ± 0.84); 3) “knowledge about disease and treatment” (4.37 ± 0.81); 4) “funeral support” (2.85 ± 1.30); 5) “information for hospice care” (3.01 ± 1.14); 6) “psychological support from patients” (3.08 ± 1.18); 7) “symptom control for patients” (4.26 ± 0.95); 8) overall (3.6 ± 0.75)
S14 [39]: Fukui S,2004 QS: 2	Japan	Inpatients	Questionnaire survey	Sampling: convenience sampling Sample size: 66 Age (yr): 55.6 ± 12.1 Gender: 46/66 (F)	Family caregivers of mixed advanced cancer patients	66/125 (52.8%)	Self-designed information needs questionnaire: 7 items
							Information needs (p. 32): Disease-related Information 1) Information on disease (54, 82%); 2) Information on treatment (48, 73%); 3) Information on prognosis (43,65%) Care-related information 1) Patients’ physical care (40, 61%); 2) Patients’ psychological care (33,56%); 3) Family care (31,47%)
S15 [40]: Dubenske LL, et al., 2008 QS: 3	USA	NR	Questionnaire survey	Sampling: convenience sampling Sample size: 159 Age (yr): 50.28 ± 12.91 Gender: 159/159 (F)	Informal female caregivers of mixed advanced cancer patients	NR	Self-designed Cancer Caregiver Needs Checklist: 9 domains and 104 items
							Information needs (p. 269): 1) Disease/ medical (0.59 ± 0.29); 2) Caregiving (0.56 ± 0.27); 3) Relating with the patient (0.59 ± 0.31) 4) Caregiver well-being (0.41 ± 0.30); 5) Financial/legal (0.28 ± 0.35); 6) Family and close others (0.42 ± 0.33) 7) Future outlook (0.42 ± 0.39); 8) Dying (0.48 ± 0.33); 9) Spirituality (0.19 ± 0.27)
S24 [49]: Mangan PA, et al. 2003 QS: 3	USA	NR	Qualitative study (focus group)	Sample size: 32 Active caregivers (*n* = 17) Bereaved caregivers (*n* = 15) Sampling: unclear	Informal caregivers of mixed advanced cancer patients (metastasis)	56/60 (93.3%)	Semi-structured focus groups interview (audiotaped) and constant-comparative for analysis
							Unmet needs (p. 247): 1) Medical care such as provision of information, coordination of care; 2) quality of life (caregiver well-being including physical and emotional, caregivers roles); 3) help from others (practical assistance and social support) 4) unsolicited needs such as non-professional information needs, impacts on their family
S26 [51]: Joad ASK, et al., 2011 QS: 2	India	Mixed	Interview survey with semi-structured questionnaire	Sampling: unclear Sample size: 56 Age (yr): 36 caregivers aged 30–60 Gender: unclear	Family caregivers 3–6 months after the death of patients (cancer sites unclear)	NR	Semi-structured questionnaire
							Unmet needs (pp. 192–193): 1) Medical needs: “lack of home -care services” (17%); “training in “care giving”” (71%); “need for an admission to a hospice/hospital” (40%). 2) Psychological needs: 1) “felling of tense” (39%); 2) “anxious” (17%); 3) “depressed” (32%); 3) Financial needs: “need financial help from other families or friends” (55.6%); 4) Information needs: “help in communicating disease status and prognosis with their loved one” (35%); 5) Social needs: “lack of social life” (71.4%); “affected the relationships and interactions with others” (42.9%)
S27 [52]: Buck HG, et al., 2008 QS: 2	USA	Home-based	Questionnaire survey	Sampling: unclear Sample size: 110 Age (yr): 64.7 ± 14.6 Gender: 83/110 (F)	Informal caregivers of mixed advanced cancer patients	NR	Spiritual Needs Inventory (SNI): 17 items
							Top 10 unmet needs of each item: 1) “be with family” (20%); 2) “laugh”(16%); 3) “be with friends”(12%); 4) “see smiles of others”(12%), 5) ‘think happy thoughts’(11%), 6) “be around children” (10%); 7) “go to religious services” (10%); 8) “talk about day-to-day things” (8%); 9) “read inspirational materials” (8%), 10) “talk with someone about spiritual issues” (6%)
S32 [57]: Carter N, et al., 2010 QS: 3	USA	Mixed	Qualitative study (semi-structured in-depth interview and focus group)	Sampling: unclear Sample size: 19 (16 wives, 3 children) Gender: unclear	Family caregivers of advanced prostate cancer	NA	Semi-structured in-depth interview (40–90 min) and focus group (60–90 min), audiotaped
							Needs (pp. 167–168): 1) informational needs regarding disease, treatment, side effects and care services, etc. 2) “uncertainty about the future” 3) caregiver burden including supporting the physical, functional and emotions needs of patients 4) “practical assistance needs like household chores” 5) “feelings of isolation as lack of social activities”
S37 [62]: Lee HT, et al., 2013 QS: 3	Taiwan	Home-based	Qualitative study (in-depth interview)	Sampling: consecutive sampling Sample size: 44	Family caregivers of terminal cancer patients (mixed cancer sites)	44/49 (89.8%)	In-depth interview with open-ended questionnaire (30–40 min) (tape recorded)
							Needs: 1) Emotional support from families and professionals including listening, encouragement, etc. 2) Information needs regarding “symptom management, nutrition, concerns about dying, medication and nursing aids” (p. 633).
S39 [64]: Chen HY, et al.,2008 QS: 2	Shanghai, China	Inpatients	Questionnaire survey	Sampling: convenience sampling Sample size: 89 Age (yr): (23–72, median 52.1) Gender: 58/89 (F)	Family caregivers of advanced cancer patients (cancer sites unclear)	89/100 (89.0%)	Self-designed questionnaire (unclear items)
							Needs (p. 19): 1) prognosis of disease (100%); 2) help to realize patient’s wishes(100%); 3) continuous support after discharge from hospital(100%); 4) knowledge of self-care(100%); 5) relevant knowledge of disease(98.9%); 6) regular counseling service (84.3%); 7) emotional support(69.7%); 8) pain management of patients(59.6%); 9) accompany (50.6%)
Studies Regarding Both Advanced Cancer Patients and their Informal Caregivers (*n* = 5)							
Author, Year & QS	Region	Setting	Study Design	Participants	Diagnosis	Response Rate	Data Collection Method/ Instrument & Findings
S23 [48]: Dehghan R, et al., 2012 QS: 4	Bangladesh	Outpatients	Qualitative study (in-depth interview)	Sampling: convenience sampling Sample size: 20 Patients (n = 3), Family members (*n* = 9), Clinical staffs (n = 8)	Advanced breast cancer and family members	NA	Semi-structured in-depth interview with open-ended questions (tape recorded) and qualitative description for analysis
							Needs (pp. 147–148): 1) “social needs of patients and families” due to financial impact, economic uncertainty and needs for social security; 2) “psychological and spiritual needs of patients and families”: feeling of sadness, anxiety, anger, abandonment, fear and hopeless; 3) “need for information among patients and families”. 4) “Access to and receipt of care from professional systems and providers”
S31 [56]: Wong RK, et al., 2002 QS: 2	Canada	Outpatients	Questionnaire survey	Sampling: unclear Sample size: 144 Patients: *n* = 71 Caregivers: *n* = 73 Age (yr): Patients: unclear Caregivers: unclear Gender: unclear	Mixed advanced cancer patients and their caregivers	144/264 (55%)	Advanced Cancer Information Needs Survey (ACIN): 22 items
							Needs for patients: 1) “pain control” (75%), 2) “weakness and fatigue” (58%), 3) “shortness of breath” (52%), 4) “what cause cancer” (48%), 5) “home care services” (46%), 6) “communicating with loved ones” (46%) Needs for caregivers: 1) “pain control” (82%), 2) “weakness and fatigue” (66%), 3) “home care services” (58%), 3) “what cause cancer” (53%), 4) “how can we prevent cancer” (58%), 5) “why are some cancers not curable” (56%)
S34 [59]: Hwang SS, et al., 2003 QS: 4	USA	Mixed	Questionnaire survey	Sampling: consecutive sampling Sample size: 100 Age (yr): (27–85, median 62) Gender: unclear	Informal caregivers of advanced cancer patients (cancer sites unclear)	100/ 149 (67.1%)	The Family Inventory of Needs (FIN): 20 items Caregiver’s Perception of Patients’ Unmet Needs (PPUN): 14 items
							Perception of Patients’ Unmet Needs (PPUN): 1) physical (80%), 2) nutritional (51%), 3) daily living (44%), 4) emotional (33%). Caregiver unmet needs (FIN): 1) “having information about what to do for the patient at home” (37%); 2) “knowing when to expect symptoms to occur” (31%); 3) “being told about people who could help with problems” (26%); 4) “knowing the probable outcome of the patient’s illness” (26%)
S38 [63]: Liu Y, 2008 QS: 3	Shanghai, China	Home-based	Questionnaire survey	Sampling: convenience sampling Sample size: 400 Age (yr): Patients:60.61 ± 12.67 Caregivers: 56.04 ± 12.57 Gender: Patients:63/115(F) Caregivers:29/113(F)	Mixed cancer patients at stage III/IV and their caregivers	228/400 (57%) (patients:115, caregiver:113)	Self-designed needs questionnaire for advanced cancer patients and their caregivers
							Needs for patients (pp. 30–31): 1) psychological: families’ understanding and support(96.5%), etc. 2) Physical care: information of treatment, rehabilitation (80.9%), etc. 3) Social: peer activities and support (54.8%), etc. Needs for caregivers (p. 38): 1) psychological: communication with families and professionals (76.1%), etc. 2) social: information about treatment and prognosis(81.4%) etc. 3) educational: medication guidance(80.5%) etc.
S42 [67]: Miu J, et al., 2016 QS: 2	Shanghai, China	Inpatients	Questionnaire survey	Sampling: unclear Sample size: 42 (42 patients and 42 family caregivers) Age (yr): Patients:72.9 ± 11.6 Caregivers: 55.9 ± 13.45 Gender: Patients:18/42 (F) Caregivers:23/42 (F)	Mixed advanced cancer patients and their caregivers	42/45 (93.3%)	Self-designed needs questionnaire for advanced cancer patients and their caregivers [63]
							Needs for patients (p. 2387): 1) “families’ understanding and support” (2.43 ± 0.59); 2) “relieving constipation” (2.38 ± 0.62) 3) “psychological support for caregivers after the death of themselves” (2.36 ± 0.66); 4) “pain assessment” (2.33 ± 0.61); 5) “pain management” (2.31 ± 0.64); 6) “improving appetite” (2.31 ± 0.6) Needs for caregivers: 1) “dietary and nutrition” (2.38 ± 0.66); 2) “guidance about how to help patients do activities” (2.38 ± 0.66); 3) “pain assessment” (2.38 ± 0.73); 4) “communication between families and professionals” (2.36 ± 0.58); 5) “information about treatment and prognosis” (2.33 ± 0.65)

Notes 1: QS: overall quality score; ADL: Activities of daily living; M: male; F: female; G1: group1; G2: group2; G3: group3; EC: Esophageal; PBC: Pancreaticobiliary; EORTC QLQ-OES18: EORTC QLQ-Esophagus (OES) 18 (Esophagus cancer module) questionnaire; EORTC QLQ-PAN26: EORTC QLQ-Pancreatic (PAN) 26 (Pancreatic cancer module) questionnaire; EORTC QLQ-C30: European Organization for Research and Treatment of Cancer Quality of Life Core 30; a: only the baseline data was used in this review

Notes 2: in the “Data Collection Method/ Instrument & Findings” column, direct quotations from several included quantitative studies using commonly utilized research scales with documented psychometric properties were details of each of the used research questionnaire items. Thus, information regarding page numbers was not provided, but that for direct quotations from studies using self-designed semi-structured questionnaires and/or qualitative methods, as well as page numbers for such quotations, was provided

### Descriptions of unmet needs in patients with advanced cancer

A total of 12 domains of unmet needs were identified from 34 quantitative and 4 qualitative studies. These domains included physical, ADL, psychological, health system and information, patient care and support, social, communication, financial, spiritual, autonomy, sexuality, and nutritional needs.

#### Unmet patient needs based on quantitative studies

Study sample sizes ranged from 40 to 977, with the average sample size being 165 and the response rate ranging from 36 to 100%. Physical needs were reported in 24 studies, and the most prominent physical unmet need was fatigue [6, 31, 33, 34, 42, 43, 45, 47, 50, 54, 56, 63]. In terms of ADL, 11 studies were included, and the most highlighted item was “not being able to do the things you used to do” [6, 33, 50, 58, 60]. Twenty-eight studies reported psychological needs, and the most common item was “emotional support” [6, 28, 29, 31, 33, 36, 41, 45, 46, 50, 70, 72, 73]. In terms of health system and information, “being informed about benefits and side-effects of treatment” was the most common one [31, 41, 42, 44, 54, 61, 63, 66, 69, 75]. With regards to patient care and support needs, two prominent unmet needs, namely, “reassurance by medical staff that the way you feel is normal” [33, 41] and “doctor acknowledges and shows sensitivity to your feelings and emotional needs” [33, 42], were identified. “Family and friends’ support” was the most common social unmet need [29, 45, 54, 55, 63, 65, 67]. Communication and financial support needs were also reported [28, 29, 31, 36, 43, 46, 54, 56, 63, 66, 55, 70]. “Meaning of death” [31, 36] was the most commonly mentioned spiritual need. “I can do less than before” [31, 34, 43] was the most prominent unmet autonomy need. Detailed unmet needs and their prevalence are presented in Table 3.

**Table 3:** Overall unmet needs domains and prevalence ranges of prominent items by each domain (Patients)

Domains	Number of studies	Subdomains/ items	Prevalence ranges
Physical	22	Fatigue	18–76.3% [6, 31, 33, 34, 42, 43, 45, 47, 51, 54, 56, 63]
		Pain	18–75% [6, 31, 33, 36, 45, 47, 50, 60, 66]
		Sleep problems	21.1–37.1% [28, 47]
		Dyspnea	19–67.3% [36, 45, 56]
		Lack of appetite	13–80% [45, 50, 55, 63]
		gastrointestinal symptoms	12–45.1% [45, 66]
		“Felling unwell a lot of the time”	17.3–44.7% [6, 33, 50]
Activities of Daily Living (ADL)	11	“not being able to do the things you used to do”	19–46.9% [6, 33, 50, 58, 60]
		“Work around the home”	18.6–44.2% [6, 33, 50, 55]
Psychological	25	“Uncertainty about the future”	21.4–62.4% [6, 31, 33, 41, 60]
		Emotional Support	10.1–84.4% [6, 28, 29, 31, 33, 36, 41, 45, 46, 50, 70, 72, 73] (Anxiety [6, 33]: 15.3–41.8%; Depression [31, 33, 41, 50]:15–62.4%)
		“worry that the results of treatment are beyond your control”	19–71.8% [6, 41, 50, 58, 60]
		“Feeling about death and dying”	32.5–62.4% [33, 41]
		“Fears about the cancer spreading”	17.6–78.8% [6, 31, 41, 42, 47, 50, 55]
		“concerns about the worries of those close to you”	27.9–68.2% [6, 33, 41, 50, 60]
		“Support in coping”	24.3–57.5% [29, 54, 55]
		“Learning to feel in control of your situation”	32.5–56.5% [33, 41]
		“Fear of physical suffering”	16.7–62.9% [31, 34, 36, 47, 50]
Social	9	family and friends’ support	9.9–96.5% [29, 45, 54, 55, 63, 65, 67]
		volunteers	18.7% [65]
Communication	5	Communication	7.7–87.9% [28, 29, 56, 63, 66]
Financial	8	Financial	6.6–72% [28, 31, 36, 43, 46, 54, 55, 70]
Spiritual	5	Meaning of death	15–85.4% [31, 36]
		Religious	44% [43]
		“being able to choose the place where you want to die”	11–15% [54, 55]
Autonomy	5	“I can do less than before”	17–83% [31, 34, 43]
		“experiencing loss of control over one’s life”	16–19% [31, 47]
Patients care and support	3	“Reassurance by medical staff that the way you feel is normal”	32.5–56.5% [33, 41]
		“doctor acknowledges and shows sensitivity to your feelings and emotional needs”	34.8–39.5% [33, 42]
Healthcare service and information	14	“Being informed about things you can do to help yourself to get well”	41–65.9% [33, 41, 42, 60, 75]
		“Having one member of hospital staff with whom you can talk to”	32–72% [33, 41, 75, 58, 75]
		“Being informed about your test results as soon as feasible”	50.8–62.5% [41, 42, 75]
		“benefit and side-effects of treatment”	4–66.7% [31, 41, 42, 44, 54, 63, 66, 69, 75]
		“Being given written information about the important aspects of your care”	42.3–52.9% [41, 75]
		“Being treated like a person not just another case”	34.5–54.1% [37, 41, 61, 75]
		“Being informed about cancer which is under control”	54.1–60.4% [41, 75]
Sexuality	4	Sexuality	5–75% [31, 36, 45, 58]
Nutrition	2	Nutrition	38.9–43.2% [28, 66]
Counseling	1		17–24% [31]

Notes: Needs items (sentences or phrases) which were put in the quotation marks were directly extracted from the corresponding included studies

#### Unmet patient needs extracted from qualitative studies

According to four qualitative studies [48, 71–73], several unmet needs that were similar to those identified in quantitative studies were extracted and categorized. For instance, patients commonly expressed “pain, fatigue or side effects of treatment, such as urinary incontinence and loss of sexual function” (p. 191–192) (physical needs) [73], “*feelings of fear, hopelessness and uncertainty about the future”* [48, 71] or “*feelings of sadness, anger, anxiety, frustration and desperation”* [48, 71, 73] (psychological and spiritual needs), “*insufficient information from professional staff”* (information needs) [48, 72, 73], “*need more social security”* (social needs) [48], and “not being regarded as a person” (p. 178) (healthcare service and information needs) [72]. However, the needs in qualitative studies were more detailed than those in quantitative studies, and the specific causes of unmet needs were identified. For example, patients elaborated that “lack of dialogue with the professionals led some patients to feel neglected and uncertain in their sense of belonging” (p. 178) [72] was the cause of “not being regarded as a person” (p. 178). Additionally, “*sadness, anger, frustration and regret”* resulted from “*some unsolved issues about diagnosis and treatment decisions”* [73]. Several unmet needs identified from the qualitative data were not identified in quantitative studies. For instance, subjects expressed “what they had achieved in their lives and what still needed to be done before death” (p. 42), “establish themselves as they ‘really’ are” (p. 41) (spiritual needs) [71], and “patients want to *be proactive* in problem solving” (p. 179), but they did not know how to do it (autonomy needs) [72].

### Descriptions of unmet needs in informal caregivers

Seven unmet need domains were extracted on the basis of qualitative (*n* = 4) and quantitative (*n* = 13) studies.

In terms of the quantitative studies, the sample size ranged from 42 to 1662, with the mean sample size being 259. The response rates ranged from 41.4 to 95.6%. Seven domains, including information, physical, psychological, financial, cancer care service, spiritual, and social needs, were identified. Information domain included two subdomains, namely, illness and treatment and care-related information. Unmet needs regarding illness and treatment information were mentioned in nine studies, and the prevalence ranged from 26 to 100% [9, 30, 35, 39, 40, 51, 56, 67, 63]. Care-related information was reported in 10 studies with the prevalence rate in the range of 21–100% [9, 30, 35, 39, 40, 51, 59, 63, 64, 67]. With regard to cancer care services, 21–72.3% of the informal caregivers presented unmet needs in terms of quality of care [29, 30, 35], and 14–100% reported unmet needs on transitional care services [30, 32, 51, 64]. The percentages of the five other domains, including physical, psychological, financial, spiritual, and social unmet needs, were 42.8% [32], 17–78.3% [32, 35, 51, 63, 64], 17–67.3% [30, 32, 35, 51], 3.8–100% [30, 32, 52, 64], and 42.9–71.4% [51], respectively. Furthermore, “managing concerns about the cancer coming back” (78.3%) [35], “finding out about financial support and government benefits for you and/or the person with cancer” (60.9%) [35], “help to realize patient’s wishes” (100%) [64], and “lack of social life” (71.4%) [51] were reported as the most common psychological, financial, spiritual, and social needs.

According to four qualitative studies [48, 49, 57, 62], three similar unmet need domains, namely, informational, psychological, and social needs, were identified through summative content analysis. Informal caregivers commonly stated about “*unmet information needs in terms of disease, treatment, side effects, care services, symptom management, nutrition, medication and nursing aids”* (informational) [48, 57, 62], “*feelings of sadness and loneliness, as well as a sense of abandonment, fear and helplessnes*s” (p. 147) [48] or “*insufficient listening and encouragement from other family members and professionals*” [62] (psychological), and “*feelings of isolation due to the lack of social activities*” (social) [57]. Several specific unmet needs, including the manner of communication between professional staff and caregivers or patients, the administration and function of the healthcare system, and some practical assistance, such as cleaning the house and walking the dog [49], were also identified in qualitative studies [49].

### Variables associated with the unmet needs of patients with advanced cancer

Variables associated with the unmet needs of patients with advanced cancer are summarized in Table 4. Relevant variables were categorized as patient-related variables (demographics, disease-related, physical, and psychological) and informal caregiver-related variables (age, gender, and psychological distress of informal caregivers).

**Table 4:** Summary of the variables associated with advanced cancer patients’ unmet needs

Study	Demographics						Physical		Disease-related			Psychosocial			Caregiver		
	Older	Female	Living alone	Married	High education	High income	Physical	ADL (dependent)	Cancer sites	Stage	Treatment	Anxiety	Depression	High QOL	Distress (anxiety/ depression)	Older	Female
[28]	+ (phy)	+ (psy, com)	+ (psy)	- (psy, com)	↔			+ (info, com, psy, occup)	↔								
[29]	- (phy, psys, com)																
[31]	-(fin, psys,)																
[33]	↔	+ (phy, ADL)		+ (phy, psys, ADL)								+(phy, psys, ADL)					
[41]												+ (psy, phy, ADL, HSIPS)		-(psy, phy, ADL, HSIPS)			
[42]	-(psy)	↔			+ (phy ADL)		+ (phy, psys, ADL)			↔	↔	+ (psy, ADL,phy, HSIPS)	-(HSIPS) +(psy)				
[43]														- (phy, psy, spiri)			
[44]	↔	↔		-(info)	+(info)						↔	+(info)	↔				
[46]	- (phy, psys, fin)	↔		↔	+ (comm- unity)	- (fin)			+/ ↔^*^								
[58]	↔			- (phy, ADL) +(sex)	+ (sex)	↔	+ (phy, ADL, psy, HSIPS)			↔	-(HSIPS)	+ (psy)	↔				
[63]	-(phy)	+ (phy)		-(phy, soc)		–			+/↔^*^						+	-(psy)	-(psy)
[70]	-(phy, fin,med)						+(phy,psy fin,med)		↔	+			+(psy,fin,med)				
[75]							+ (HSIPS, psy, phy, ADL)			↔	+ (psy)						

Notes: “-”: negative relationship; “+”: positive relationship; “↔”: no significant relationship; “*”: relationship variable across different types of cancer; “fin”: financial needs; “PM”: pain management; “soc”: social needs; “phy”: physical needs; “psy”:psychological needs; “psys”: psychosocial needs; “inf”: information needs; “com”: communication needs; “occup”: occupational needs; “HSIPS”: health system, information, and patient care support; “med”: medical needs; “spiri”: spiritual needs

In several studies, age, gender, marital status, education level, and income level were insignificantly associated with patients’ unmet needs. Although a significant relationship was reported, results were inconsistent across studies in terms of age and marital status. With regards to gender, three studies [28, 33, 63] reported that female patients indicated more physical and psychological unmet needs than those of male patients. Patients who were living alone experienced high psychological needs [28], and patients with high educational level presented considerable unmet needs in physical [42], ADL [42], information [44], community service [46], and sexuality [58] domains. Moreover, financial needs were less reported in patients with high income [46, 63].

Four studies [42, 58, 70, 75] explored the relationships between symptom distress and unmet needs, and all these studies showed that patients with symptom distress experienced more unmet needs in the psychological, physical, and ADL domains. Patients with poor ability in daily living [28] indicated more unmet needs than those of independent patients, especially in terms of information, communication, psychological, and occupational needs.

Two studies [28, 70] showed that no relationships were observed between cancer site and their unmet needs, but two other [46, 63] studies showed opposite results. Two [42, 75] out of five studies reported that no relationship was observed between cancer stage (only stages III and IV) and unmet needs, and three ones [58, 63, 70] indicated that patients with stage IV cancer presented more unmet needs than those with stage III cancer. Results were inconsistent across studies for cancer treatment, with two studies showing no relationship [42, 44] and two other studies suggesting either positive [75] or negative [58] relationship.

Patients with anxiety experienced high levels of physical, psychological, healthcare, and information, as well as ADL unmet needs, which was confirmed across several studies [33, 41, 42, 44, 58]. Patients with depression [42, 44, 58, 70] demonstrated varied results. Patients with low quality of life showed high unmet needs, especially in physical and psychological domains [41, 43]. Patients reported more unmet needs when their caregivers were male [28], young people [28], or those who suffered from psychological distress [28].

### Variables associated with the unmet needs of informal caregivers

Older caregivers [30, 35] showed less unmet needs in terms of financial, social, and care-related information needs than those of younger caregivers. Caregivers in different caregiving settings reported different levels of unmet needs (home>general hospital>hospice care unit) [32, 39]. Caregivers with many physical problems experienced many unmet needs [35, 63]. Caregivers had higher levels of unmet needs when patients suffered from anxiety [35], depression [35], or low physical performance [35]. Results varied across studies in terms of gender [30], length of caregiving [9, 63], and education level of caregivers [63] (Table 5). Similarly, results were conflicting with regard to the relationships between caregivers and patients. One study [39] showed that spousal caregivers presented many information needs, and another study [63] indicated that non-spousal caregivers reported many unmet needs.

**Table 5:** Summary of the variables associated with informal caregivers’ unmet needs

Study	Demographics of caregivers					Caregivers’ physical symptom	Relationship	Patients-related		
	Older	Female	Education level	Length of caregiving	Care setting		Spousal caregivers	Patients’ anxiety	Patients’ depression	Lower physical performance
[30]	-(fin, PM, soc.,)	F (+phy) M (+ inf)								
[32]					Conventional hospital care > hospice care (symptom management, psy support, religious support)					
[35]						+ (overall)		+(overall)	+(overall)	+(overall)
[9]				–						
[39]	–				Home > hospital (inf)		+			
[63]	-(soc,psy,inf)		-(psy) +(soc)	+(soc)		+	-(inf)			

Notes: “-”: negative relationship; “+”: positive relationship; “fin”: financial needs; “PM”: pain management; “soc”: social needs; “phy”: physical needs; “inf”: information needs; “overall”: overall needs

### How their unmet needs were assessed in the included studies

For patients with advanced cancer, the most commonly used multidimensional instruments were Supportive Care Needs Survey (SCNS, *n* = 8) [6, 33, 41, 50, 58, 60, 61, 75], Problems and Needs in Palliative Care questionnaire (PNPC, *n* = 5) [31, 34, 36, 44, 47], and Needs Assessment of Advanced Cancer Patients (NA-ACP, *n* = 3) [38, 54, 55]. Other multidimensional instruments that were adopted included Cancer Needs Questionnaire [42], Patient Needs Assessment in Palliative Care [43], 3-Levels-of-Needs Questionnaire [45], Needs Assessment Tool: Progressive Disease–Cancer [68], Caregiver’s Perception of Patients’ Unmet Needs [59], and other instruments without reporting their psychometric properties. Among studies that focused on one specific need domain (*n* = 4), three explored information needs [44, 56, 69], and one investigated spiritual needs [37]. The unidimensional instruments adopted included the following: Toronto Information Needs Questionnaire [69], Advanced Cancer Information Needs [56], PNPC (only used the items of the information domain) [44], and an instrument [37] for spiritual needs assessment without specifying its psychometric properties. Overall, more than half of the quantitative studies (20/34) adopted instruments with acceptable validity and reliability.

Among the 13 quantitative studies reporting unmet needs of informal caregivers, comprehensive unmet needs (multiple domains) were explored in 10 studies [9, 30, 32, 35, 40, 51, 59, 63, 64, 67]. Different quantitative studies used different measures, which included PNPC questionnaire-caregiver form [30], Family Inventory of Needs [59], Partners and Caregivers SCNS [35], needs of family caregivers of patients with advanced cancer [9], and other self-designed instruments [32, 40, 51, 63, 64, 67]. Among the three other studies that focused on unidimensional needs assessment, two [39, 56] measured information needs, and one [52] explored spiritual needs. The scales used were Spiritual Needs Inventory [52] and two other self-designed instruments, namely, with [56] or without [39] psychometric property testing. Among all the 13 studies, only four studies used scales with documented psychometric properties.

## Discussion

The included studies highlighted that both advanced cancer patients and their informal caregivers possess a wide range of unmet needs. Psychological and physical unmet needs are two areas of focus for patients with advanced cancer; this result is consistent with a previously published review [7]. Among informal caregivers who had experience in managing patients’ negative emotions, more than 30% of them reported that emotional management is the most challenging part of caregiving [76]. Three other unmet needs, namely, the need for autonomy, communication, and nutrition, were identified in this review compared with the previous review [7]. These needs may be related to the differences in cultural contexts, healthcare systems, and economic levels because several included studies in this review were conducted in eastern and developing countries. For instance, the need for autonomy is commonly culture-related [36]. Family members usually make decisions for patients in eastern cultures because family-collective decision-making is much more popular there than in other cultures [77]. This result showed the importance of developing tailored healthcare services or interventions based on context-specific unmet needs. Disease-related information needs were the most commonly reported unmet needs of informal caregivers. Considerably fewer studies reported unmet needs that are associated with the caregivers’ own well-being, as they generally focus more on the patients’ well-being than their own [30]. The prominent care needs of each domain were identified for patients with advanced cancer and informal caregivers in this review provide useful information and evidence for the development and implementation of tailored healthcare services. For example, emotional support was identified as the most commonly unmet need in the psychological domain for patients, thereby indicating that emotional distress (e.g., anxiety and depression) management should be a priority when providing mental health services. In addition, patients with advanced cancer and informal caregivers’ unmet need domains involved multiple disciplines, which indicated that healthcare services should be multidisciplinary. The value of multidisciplinary care for patients with cancer has been well recognized [78]. Support for informal caregivers is suboptimal in many instances [79]. The unmet needs of informal caregivers are often ignored and excluded from healthcare planning [80, 81].

The prevalence of unmet needs varied across the quantitative studies for both patients and caregivers. This variability may be caused by the heterogeneity of the included studies, which were conducted within different cultural contexts, healthcare systems, and economic levels that may be associated with unmet needs. High-income countries or regions generally present well-established healthcare service systems, which can facilitate the timely identification and resolution of healthcare problems (several physical symptoms particularly require high-quality professional support [28]). Different study designs, especially the diverse instruments used, for unmet needs assessment also contribute to this heterogeneity. The highlighted heterogeneity makes it difficult to gauge and pool the percentages of unmet needs by domains. SCNS was the most commonly used instrument, which was used in eight studies. However, these eight studies adopted five different variants of the same scale, with 13 [61], 33 [58], 34 [6, 33, 41], 59 [60], and 61 items [50] for each of the five versions. Different methods of need classification are also a major barrier in gauging unmet needs by domains. For instance, in SCNS, several items were classified as spiritual needs (e.g., [50]). In other studies, the same items were coded as psychological needs (e.g., [41]). Moreover, approaches in defining unmet needs were inconsistent. Among studies that utilized the SCNS, several of them regarded moderate and high levels of need as unmet needs (e.g., [41]. In other studies, low need level was calculated as an unmet need (e.g., [50]). Different reporting methods also caused heterogeneity. Several studies reported the prevalence of unmet needs by domains without specifying the percentage of items within each domain. Some studies (e.g., [33]) only listed the prevalence of the top 10 or 20 items without reporting the prevalence by domain. Thus, directly combining the prevalence of reported items within a domain may increase the risk of overestimating the actual unmet need level [21].

Although consistent results across studies showed that patients with advanced cancer with symptoms of distress and anxiety and low quality of life are more likely to report high demands of unmet needs, the conclusion must be interpreted with caution. Causality cannot be established because almost all of the included studies were cross-section in design. Other patient-related variables with inconsistent results, (e.g., gender, marital status, education level, cancer site, and depression) may be caused by cultural differences and/or methodological flaws (e.g., insufficient sample size to explore relationships between two factors) of the included studies. Hence, more longitudinal studies with rigorous study designs should be adopted. In addition, whether caregivers’ health outcomes were associated with the unmet needs of patients is still unclear because of the limited evidence that can be drawn from current studies. Therefore, more studies should focus on caregiver-related variables. Relevant studies regarding variables associated with informal caregivers’ unmet needs are limited, and no conclusion can be drawn from the current findings.

Patients with cancer at an advanced stage commonly experience fluctuating unmet needs over time due to rapid disease progression [6]. Nevertheless, little is known about how patients with advanced cancer and/or their informal caregivers’ unmet needs change across the illness trajectory. Almost all the included quantitative studies investigated unmet needs at a single time point with cross-sectional study designs. Unmet care needs assessment in the majority of the included studies is also mainly problem-oriented from a biomedical lens. Few studies considered contextual issues (sociocultural and healthcare service provisions) when assessing and interpreting results in a given context although it will be of benefit to the development and implementation of tailored interventions at a local level. Accordingly, qualitative studies are an appropriate approach because it can explore participants’ in-depth experience and subjective feelings that cannot be measured by quantitative methods; additionally, the scope can be much broader than those of quantitative methods [82, 83]. Deeper understanding of unmet needs can be extracted from the qualitative studies than from quantitative findings. However, limited studies adopted qualitative study designs, and only few studies utilized mixed methods. Care needs should be comprehensively evaluated from all stakeholders, including patients, caregivers, and healthcare providers [84]. A comprehensive understanding of both patients with advanced cancer and informal caregivers’ unmet needs can enable healthcare providers to develop evidence-based and tailored interventions [18]. Nevertheless, the majority of the included studies assessed patients’ unmet needs only, and almost all included studies examined unmet needs from the participants’ own perspective rather than from the perspectives of all relevant stakeholders. Despite that the concept of patient-and-family-centered care is advocated by the WHO [16], structured unmet needs assessment of informal caregivers is still an uncommon practice. Only a few studies assessed the unmet needs of patients and informal caregivers, and their unmet needs were assessed separately. The mechanism of integrating the data of patients and caregivers should be considered to further embody the conceptualization as a whole unit. Focused group with mixed samples, including patients and informal caregivers in the same group, may be an appropriate approach. Finally, research instruments used for needs assessment in several included studies were inappropriate. Some scales are generic ones used for supportive care needs assessment. Several items, such as “fear about the cancer spreading,” may be unsuitable for patients with advanced cancer.

A strength of this systematic review is that a large number of studies with considerable information were assimilated and analyzed through a systematic method, which can minimise biases and facilitate  reliable conclusions. This work is the first systematic review conducted by considering patients with advanced cancer and their informal caregivers as a whole unit. However, this review also presents several limitations. First, subgroup analysis in terms of contexts and economic levels was not conducted. Second, given the confounding factors and insufficient number of studies in each subgroup, meta-analysis was also not performed to compare the prevalence of each identified need domain. Third, language bias cannot be excluded because only papers that were published in English or Chinese language were included. Finally, instruments for needs assessments were only summarized from the included studies, and studies in terms of instrument development were excluded.

## Conclusions

A wide range of unmet care needs existed in both advanced cancer patients and informal caregivers. Given the context-bound feature, their unmet needs should be comprehensively assessed and interpreted from the perspectives of all stakeholders within a given context by using rigorous mixed methods research and longitudinal research with prospective study designs. Assessing unmet care needs by viewing patients with advanced cancer and their informal caregivers as a whole unit is highly desirable. Associated factors of their unmet needs should not be ignored, which can provide evidence for decision-making with regards to healthcare resource allocation. The value of better examining unmet needs and their associated factors in advanced cancer patients and informal caregivers ultimately depends on how well it could inform the development and implementation of tailored healthcare service or intervention.

## Abbreviations

3LNQ: 3-Levels-of-Needs Questionnaire
ACIN: Advanced Cancer Information Needs
ADL: Activities of Daily Living
FIN: The Family Inventory of Needs
MMAT: Mixed Methods Appraisal Tool
NA-ACP: Needs Assessment of Advanced Cancer Patients
NAT: PD-C: Needs Assessment Tool: Progressive Disease-Cancer
PNAP: Patient Needs Assessment in Palliative Care
PNPC: Problems and Needs in Palliative Care questionnaire
PPUN: Caregiver’s Perception of Patients’ Unmet Needs
SCNS: Supportive Care Needs Survey
SCNS-P&C: Partners and Caregivers supportive care needs survey
SNI: Spiritual Needs Inventory
TINQ-BC: Toronto Information Needs Questionnaire

## Footnotes

1 In this review, patients with advanced cancer are defined as follows: patients with advanced, secondary, metastatic, or terminal cancer [23, 24] or patients with cancer at stage III or IV according to TNM Staging System or Dukes’ D according to Dukes’ staging system [23, 24]).

2 In this review, when external help for patients and informal caregivers’ existing problems was inadequate, any level of need for addressing the unsolved problems was regarded as unmet care needs, which included low, moderate, and high needs.

3 In this paper, direct quotations from several included quantitative studies using commonly utilized research scales with documented psychometric properties were details of each of the used research questionnaire items. Thus, information regarding page numbers was not provided, but that for direct quotations from studies using self-designed semi-structured questionnaires and/or qualitative methods, as well as page numbers for such quotations, was provided.
